# A de novo ANK1 mutation in a childhood hereditary spherocytosis: a case report

**DOI:** 10.1186/s12887-023-04092-0

**Published:** 2023-05-29

**Authors:** Yafeng Wang, Linlin Liu, Dandan Liu, Wei Liu

**Affiliations:** 1 Department of Hematology and Oncology, Children’s Hospital Affiliated to Zhengzhou University, Henan Children’s Hospital, Zhengzhou Children’s Hospital, No.33 Longhuwaihuan East Road, Zhengzhou, 450018 China; 2 Henan Provincial Key Laboratory of Children’s Genetics and Metabolic Diseases, Children’s Hospital Affiliated to Zhengzhou University, Zhengzhou, 450018 China; 3 Department of Electrocardiogram, Children’s Hospital Affiliated to Zhengzhou University, Henan Children’s Hospital, Zhengzhou Children’s Hospital, Zhengzhou, 450018 China

**Keywords:** Hereditary spherocytosis, Children, ANK1, Mutation, Biliary obstruction

## Abstract

**Background:**

Due to the heterogeneity of the phenotype of Hereditary spherocytosis (HS) patients, some patients may have rare clinical complications such as biliary obstruction and ultra-high bilirubinemia.

**Case presentation:**

A 8-y-old boy presented to the emergency with complaints of anemia for 6 years and worsened abdominal pain and scleral yellowing of the skin for 2 days. Physical examination showed tenderness in the middle and upper abdomen and splenomegaly. Abdominal CT revealed biliary obstruction. Genetic analysis revealed a de novo mutation in the gene *ANK1,* HS with biliary obstruction was diagnosed. The surgery of bile duct exploration and T-tube drainage, and splenectomy were performed successively. This patient was followed up for 13 months after splenectomy, and his condition was stable.

**Conclusion:**

The diagnosis of HS is not clinically difficult, and once a patient with HS is diagnosed, regular follow-up management and standardized treatment are required. Genetic testing is also needed to screen for other genetic disorders that may co-exist in patients with HS who do not have a good efficacy or who have a long-term chronic onset of jaundice.

## Background

Hereditary spherocytosis (HS) is a common chronic non-immune hemolytic disease [1], and the clinical features of HS are mainly jaundice, splenomegaly and hemolytic anemia, but due to the heterogeneity of the phenotype of HS patients, anemia can be mild to severe in clinical manifestations, leading to possible misdiagnosis or delayed diagnosis in some patients with atypical symptoms [2]. *ANK1* gene mutation is one of the most common causes of HS. The authors present a case of HS with a de novo* ANK1* mutation combined with biliary obstruction in a child. 

## Case presentation

An 8-y-old boy presented to the emergency department with complaints of anemia for 6 years and worsening abdominal pain and scleral yellowing of the skin for 2 days. This patient was diagnosed with HS 6 years ago at a local hospital by routine blood count, bone marrow and peripheral blood cell morphology, and erythrocyte permeability fragility test, and then was treated with red blood cell infusion and discharged. This boy has not followed up regularly in recent years. Two days ago, this child suddenly developed worsened abdominal pain and scleral yellowing of the skin, this patient had no fever, cough, sputum, blood in the stool and other symptoms. The parents denied that their son had any history of infectious diseases, food or drug allergies, genetic or congenital disorders. On physical examination, the patient’s face was pale and yellow, with severe yellowing of the skin and sclera. The abdomen was soft, with mid-upper abdominal tenderness and no rebound pain. The spleen was enlarged to approximately 5 cm below the left ribcage and was slightly hard to palpate.

**Fig. 1:**
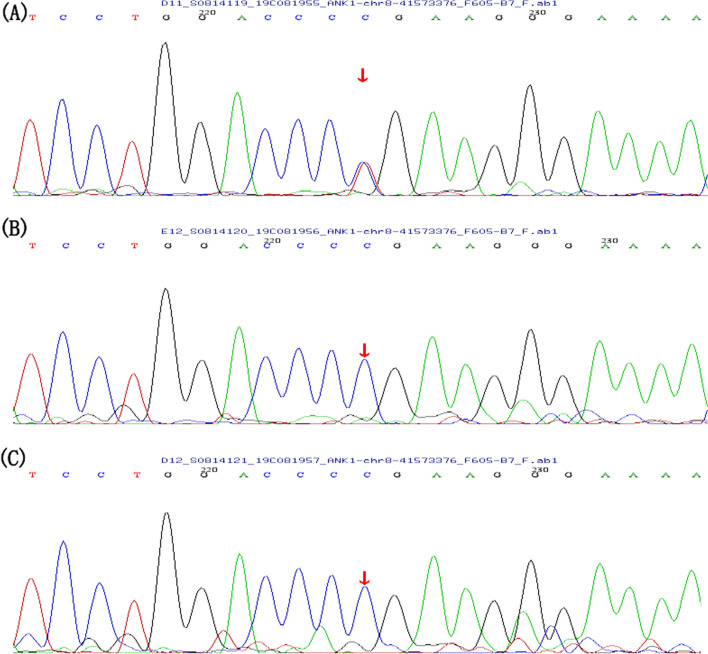
Sanger sequencing identified an ANK1 (c.1405-9G > A) mutation in this patient (**A**), his father (**B**) and mother (**C**) An arrow indicates the mutation site

**Fig. 2:**
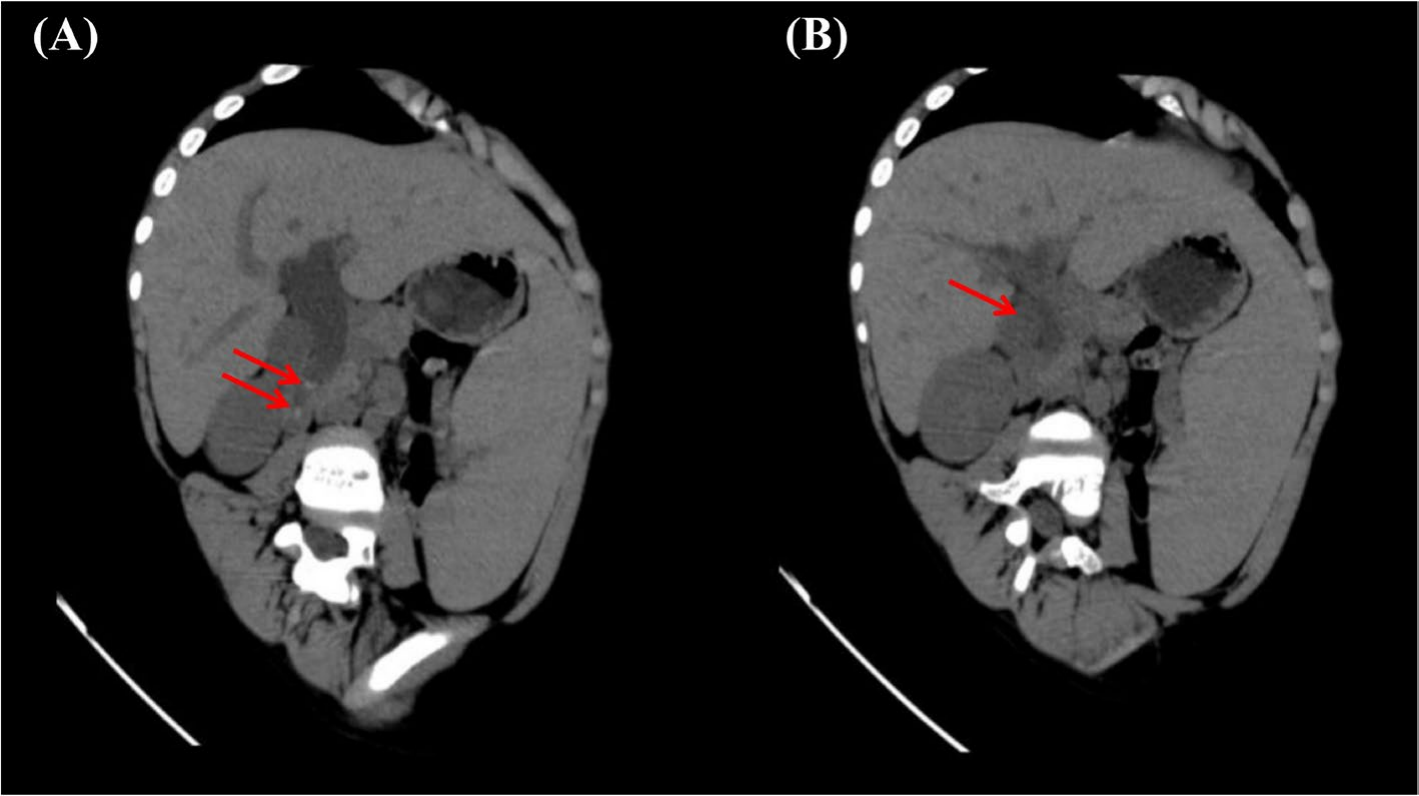
CT scan findings of the abdomen for this patient. **A** High-density shadow of the lower part of the common bile duct shown by the red arrow is considered to be gallstones. **B** Dilated intrahepatic bile duct shown by the red arrow

The complete blood count showed total leukocyte count: 7.86 × 10^9^/L, hemoglobin: 57 g/L, and spherical red blood cells accounted for 15% of peripheral mature red blood cells. The erythrocyte permeability fragility test was positive. Reticulocyte count: 645.83 × 10^9^/L (normal range 17 × 10^9^/L-70 × 10^9^/L). Total bilirubin: 679.2 μmol/L (normal range 0–20 μmol/L); conjugated bilirubin: 447.6 μmol/L (normal range 0–5 μmol/L); unconjugated bilirubin: 231.6 μmol/L (normal range 0–19 μmol/L). Genetic analysis revealed a de novo mutation in the *ANK1* gene (c.1405-9G > A, splicing mutation in exon14, Fig. 1) and no abnormalities in the *UGT1A1* gene. Abdominal CT showed biliary obstruction that obstructed the bile flow (Fig. 2), confirming the diagnosis of biliary obstruction in this pediatric HS.

After the surgery of bile duct exploration and T-tube drainage, the patient’s abdominal pain and the jaundice gradually resolved, but the bilirubin level was only slightly lower than before (hemoglobin: 71 g/L, total bilirubin: 534.2 μmol/L; conjugated bilirubin: 318.3 μmol/L; unconjugated bilirubin: 215.9 μmol/L). As the patient’s unconjugated bilirubin level was still high and associated with hypersplenism, there was still a risk of causing biliary obstruction. After completion of vaccination, splenectomy was performed 40 days after the first surgery, and postoperative infection prophylaxis and symptomatic treatment were given. This child is now 13 months post-splenectomy and is still being followed in the stable condition (the bilirubin level of 1 year after the splenectomy: hemoglobin: 134 g/L, total bilirubin: 15.5 μmol/L; conjugated bilirubin: 4.1 μmol/L; unconjugated bilirubin: 11.4 μmol/L).

## Discussion and conclusions

Biliary obstruction and hyperbilirubinemia are atypical signs of HS in children [3]. In this case, the child was clinically diagnosed with HS at the local hospital 6 years ago, but because the child’s symptoms were mild at that time, he was not followed up regularly. When the child presented with sudden worsening of jaundice and severe abdominal pain, at which time he developed potentially life-threatening complications due to biliary obstruction caused by gallstones formed by long-term chronic hemolysis. Gilbert’s syndrome (GS) is the most common form of familial unconjugated hyperbilirubinemia and can lead to hyperbilirubinemia due to reduced activity of uridine diphosphate-glucuronosyltransferase 1A1 (UGT1A1) [4]. Patients with both HS and GS have been reported to have a 4 to fivefold increased risk of gallstone disease compared with HS patients [5]. Although there is no biological link between HS and GS, both disorders may occur together in a significant number of patients [6]. In this case, the genetic testing showed mutations in the *ANK1* gene and no abnormalities in the *UGT1A1* gene, suggesting that this child lacks genetic evidence of GS. Although this case did not combine with GS, genetic testing is still needed to screen for other genetic disorders that may co-exist in patients with HS who do not have a good efficacy or who have a long-term chronic onset of jaundice.

Mutations in *ANK1* were the most common cause of HS, and at least 60 mutations have been found in *ANK1* in HS patients, including 7 missense mutations [7]. Recently, mutations occurring in different regions of the *ANK1* gene have been reported to cause clinical phenotypes, and the same mutated genes and sites may also show variation in clinical phenotypes [8]. In a Canadian cohort study, the authors reported that four children with HS carried the *ANK1* mutation (c.1405-9G > A), three of which were inherited (autosomal dominant) and one was a de novo mutation, but these four patients with HS also carried other mutated gene sites at the same time [9]. In addition, the HS patients in this article had mild anemia and slightly elevated level of unconjugated bilirubin at median age of 13 years (8.6–17.6 years), whereas in the current HS case we reported, the age of onset was younger, and the case had more severe anemia and significantly elevated unconjugated bilirubin level, resulting in gallstones and biliary obstruction [9]. Although the mutation site of the mutated genes in HS are the same, the clinical phenotypes may vary greatly and this may be related to age and race [10].

Since abnormal spherical red blood cells are mainly captured and destroyed in the spleen, splenectomy is the preferred treatment for patients with moderate to severe hemolytic HS [11]. In this case, this patient also had moderate anemia persistent hyperbilirubinemia after T-tube drainage. Although symptoms of biliary obstruction had resolved, excessive hemolysis due to HS may leading to hyperbilirubinemia, so splenectomy was performed and finally achieved satisfactory efficacy. However, regular follow-up is still required to prevent possible postoperative complications and to monitor the immune function in this patient.

In this report, we identified a de novo mutation in the gene *ANK1* (c.1405-9G > A) mutation in a Chinese patient. The diagnosis of HS is not clinically difficult, and once a patient with HS is diagnosed, regular follow-up management and standardized treatment are required. Genetic testing is also needed to screen for other genetic disorders that may co-exist in patients with HS who do not have a good efficacy or who have a long-term chronic onset of jaundice.

## Data Availability

The original contributions presented in the study are included in the article, further inquiries can be directed to the corresponding author.
